# Case Report: A Primordial odontogenic tumor

**DOI:** 10.12688/f1000research.14735.1

**Published:** 2018-05-09

**Authors:** Hatem Amer, Layla Hafed, Sally Ibrahim

**Affiliations:** 1 Department of Oral and Maxillofacial Pathology, Faculty of Dentistry, Cairo University, Cairo, Egypt; 2 Deparment of Oral and Maxillofacial Pathology, Faculty of Dentistry, El-Fayoum, El-Fayoum, Egypt

**Keywords:** Primordial, Mixed odontogenic tumor, Jaw tumor, Odontogenic.

## Abstract

**Introduction:** Primordial odontogenic tumors are a rare recently described mixed odontogenic tumor composed histopathologically of dental papilla like tissue and enamel organ like tissue. Only nine cases have been documented worldwide and we are reporting the tenth case which is from Egypt.

**Clinical finding: **A 2-year-old Egyptian boy that presented with an asymptomatic swelling of the mandible which appeared with multilocular radiolucency associated with an impacted developing tooth on a computerized tomography (CT) scan.

**Diagnoses, interventions, and outcomes** **:** The lesion was excised and diagnosed as a primordial odontogenic tumor. The patient was followed up for two years with no recurrence.

**Conclusion:** Differentiation of primordial odontogenic tumors from other odontogenic tumors, which resemble it histopathologically is crucial to avoid unnecessary aggressive treatment.

## Introduction

Primordial odontogenic tumor (POT) is a recently described mixed odontogenic tumor described in the last WHO classification of head and neck tumors ^1^. This tumor has been described as other entities in the past, because of its histological similarity to other odontogenic tumors as ameloblastic fibroma, odontogenic myxoma, and odontogenic fibroma and hyperplastic dental follicles ^2^.

Mosqueda-Taylor *et al.* ^3^ described and denominated this novel lesion which did not fulfil the criteria of any of the previously classified odontogenic tumors by reporting the clinicopathological and immunohistochemical features of six cases diagnosed as primordial odontogenic tumor.

Primordial odontogenic tumors are characterized histologically by a variably cellular loose fibrous tissue with areas similar to the dental papilla, covered by cuboidal to columnar epithelium that resembles the internal epithelium of the enamel organ, surrounded at least partly by a delicate fibrous capsule ^1^.

Only nine cases have currently been reported, and we report an additional case of an primordial odontogenic tumor from Egypt.

## Case report

**Figure 1.**
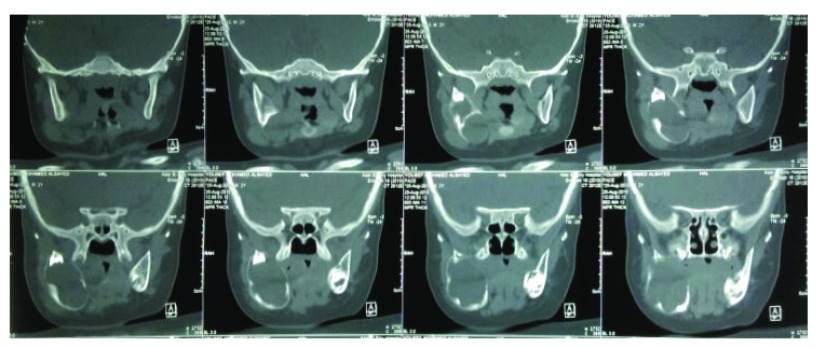
Preoperative computerized tomography scan revealed an osteolytic multilocular radiolucency at posterior mandible associated with an impacted developing tooth.

A 2-years-old Egyptian boy referred to Department of Oral and Maxillofacial Surgery, Faculty of Dentistry, Cairo University in November 2015 with a fleshy swelling arising from site of marsupialization performed two months previous. By taking patient’s history we established the lesion arose as a painful swelling covered with normal mucosa causing obliteration of the vestibule with two months duration. Manual examinations of the regional lymph nodes were negative on examination. By computerized tomography (CT) scan, a multilocular radiolucent lesion was seen associated with an impacted developing tooth in the mandibular posterior area measuring 3cm × 4cm ( Figure 1).

**Figure 2.**
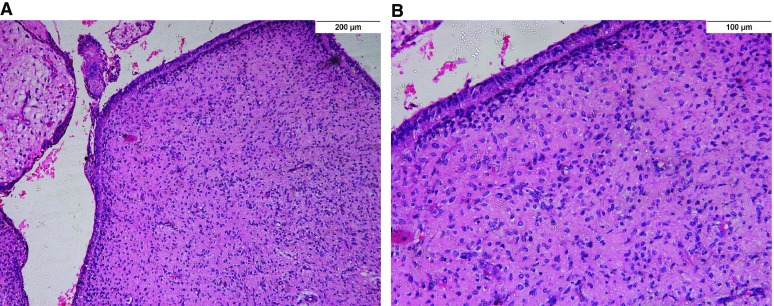
A photomicrograph of Hematoxylin and eosin (H&E) stained sections showing primitive connective tissue stroma covered by columnar epithelium, A (×100) and B (×200).

**Figure 3.**
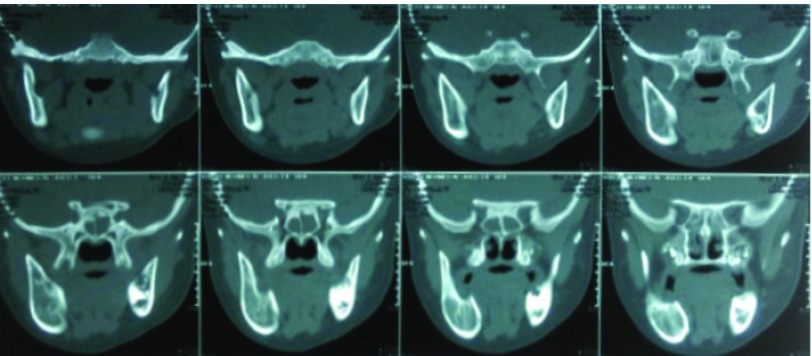
Follow up Computerized tomography scan revealed a new spongy bone formation at the site of preexisting lesion.

On aspiration, straw cystic fluid was noted. Complete surgical excision of the lesion with the impacted tooth was performed. And the excised lesion was sent to the Department of Oral and Maxillofacial Pathology, Faculty of Dentistry, Cairo University. The gross specimen showed a cystic lesion which showed areas of thickening. Hematoxylin and eosin stained sections revealed surface columnar and cuboidal epithelium covering a loose and myxoid fibrous tissue ( Figure 2) and this specimen was diagnosed as a primordial odontogenic tumor. The patient was followed up for two years with no recurrence, and new bone formation was detected in the follow up radiographs ( Figure 3).

Raw histological image A photomicrograph of Hematoxylin and eosin (H&E) stained sections showing primitive connective tissue stroma covered by columnar epithelium, (×200) Click here for additional data file. Copyright: © 2018 Amer H et al. 2018 Data associated with the article are available under the terms of the Creative Commons Zero "No rights reserved" data waiver (CC0 1.0 Public domain dedication).

## Discussion

POT is a new entity first reported in a case series of 6 cases in 2014 described as benign mixed odontogenic tumor by Mosqueda-Taylor *et al.* ^3^. Then another two cases were reported in 2015 and 2017 by Slater *et al.* ^4^ and Ando *et al.* ^5^ respectively then in 2018 Bajpai and Pardhe ^6^ described another case. This novel lesion was added to the new WHO classification of odontogenic tumors ^1^.

Table 1 shows the clinicopathological and radiographic data of the nine documented cases. All reported patients were of young age group, ranging from 3–19 years with almost equal sex predilection and with posterior mandible as predominate site. All of these clinical finding are similar to this reported case.

**Table 1.** Clinicopathological and radiographic data of the nine documented cases of primordial odontogenic tumor. M: Male; F: Female; RL: Radiolucent; UL: Unilocular; ML: Multilocular; mm: millimeter.

Study	Age	Gender	Site	Clinical Picture	Radiographic Picture	Treatment and Follow-up
**Mosqueda-Taylor *et al.*^3^**	18 years	M	Posterior mandible	Asymptomatic buccal swelling	RL, UL, well defined, 45 × 40 mm	Enucleation, 20 years, uneventful
**Mosqueda-Taylor *et al.*^3^**	16 years	M	Posterior mandible	Asymptomatic, buccal and inferior mandibular cortical bone expansion.	RL, UL, well defined, 55 × 50 mm	Lost to follow-up
**Mosqueda-Taylor *et al.*^3^**	16 years	M	Posterior mandible	Asymptomatic buccal swelling	RL, UL, well defined, 65 × 50 mm,	Enucleation, 10 years, uneventful
**Mosqueda-Taylor *et al.*^3^**	3 years	F	Posterior mandible	Asymptomatic buccal and lingual bony expansion.	RL, biloculated, well defined, 90 × 70 mm	Enucleation, 9 years, uneventful
**Mosqueda-Taylor *et al.*^3^**	13 years	F	Posterior mandible	Asymptomatic buccal swelling.	RL, biloculated, well defined, 80 × 50 mm.	Enucleation, 3 years, uneventful
**Mosqueda-Taylor *et al.*^3^**	3 years	F	Posterior maxilla	Asymptomatic buccal and palatal bony swelling.	RL, UL, well defined, 35 × 30 mm.	Enucleation 6 months, uneventful
**Slater *et al.*^4^**	19 years	M	Posterior mandible	Asymptomatic buccal and lingual bony swelling	RL, UL, well defined, 25 × 19 mm	Enucleation, 7 months uneventful
**Ando *et al.*^5^**	8 years	F	Posterior maxilla	Asymptomatic, buccal swelling	RL, UL, well-defined, 16 × 15 mm	Enucleation, 16 months, uneventful
**Bajpai and Pardhe ^6^**	17 years	M	Posterior mandible	Asymptomatic buccal swelling	RL, ML, well defined, 30 × 20 mm.	Enucleation, 6 months, uneventful

All reported lesions were expansile and asymptomatic which are opposite to our case as it was painful during presentation, which may be the result of the previous marsupialization.

 Radiographically, POT presents with a well-defined radiolucent lesion, either unilocular or bilocular, except in the case of Bajpai and Pardhe ^6^ who reported a case that appeared mulitlocular.

All documented cases shared similar histopathological criteria proposed by Mosqueda-Taylor *et al.* ^3^, as did our present case, where loose and myxoid connective tissue stroma resembles the dental papilla covered by columnar epithelium of a single layer, with the epithelium resembling the inner enamel epithelium.

Regarding the treatment approach, all previously documented lesions were treated with enucleation with different periods of follow up and reported no recurrence, in line with our presented case.

In conclusion, this is the first report case of POT from Egypt after it was defined in the latest WHO classification. Differentiation between POT and other closely resembling odontogenic tumors is crucial, especially in the case of odontogenic myxomas as it is a more aggressive tumor and requires more aggressive treatment.

The clinical, radiographical and histopathologic data of the nine previously documented cases in addition to our case will be useful to differentiate this new tumor from other odontogenic tumors, which resemble it histopathologically, to avoid unnecessary aggressive treatment modalities.

## Consent

Written informed consent for publication of clinical details and images was obtained from the patient's parent.

## Data availability

The data referenced by this article are under copyright with the following copyright statement: Copyright: © 2018 Amer H et al.

Data associated with the article are available under the terms of the Creative Commons Zero "No rights reserved" data waiver (CC0 1.0 Public domain dedication). 

Dataset 1: Raw histological image. A photomicrograph of Hematoxylin and eosin (H&E) stained sections showing primitive connective tissue stroma covered by columnar epithelium, (×200). 10.5256/f1000research.14735.d202214 ^7^
