# Supplementary figures and images for: Case Report: A Primordial odontogenic tumor
Source: F1000Res. 2018 May 9;7:562. [Version 1] doi: 10.12688/f1000research.14735.1 (PMC5989144; doi:10.12688/f1000research.14735.1)

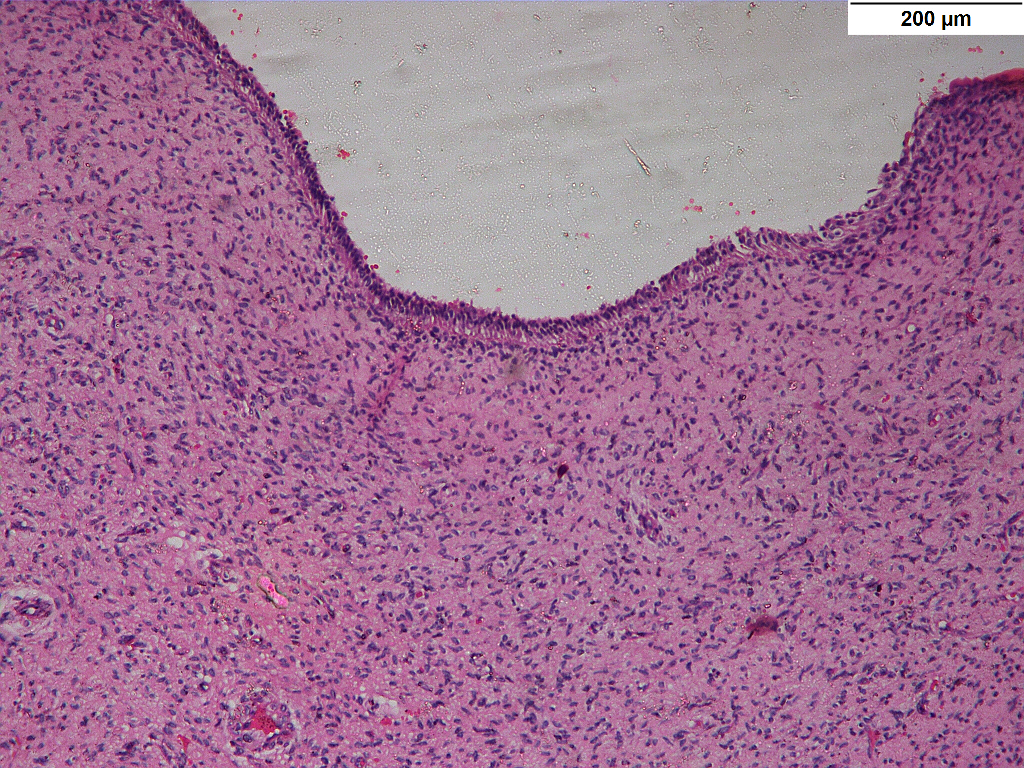

Supplement: Raw histological image — A photomicrograph of Hematoxylin and eosin (H&E) stained sections showing primitive connective tissue stroma covered by columnar epithelium, (×200) [file f1000research-7-16032-s0000.tgz › 3f2d0f6e-d952-4be7-9306-9f1af02e3a9f_Additional_raw_figure.tif]
