# Supplementary material for: Case Report: A Primordial odontogenic tumor
Source: F1000Res. 2018 May 9;7:562. [Version 1] doi: 10.12688/f1000research.14735.1 (PMC5989144; doi:10.12688/f1000research.14735.1)
Supplement: Peer review history

# Peer review: Referee response for version 1

**DOI:** 10.5256/f1000research.16032.r34184

**Published:** 2018-07-12

**Authors:** Manal Al-Hajri

**Affiliations:** 1 Oral Medicine, Oral Diagnosis and Periodontology Department, Sana'a University, Sana'a, Yemen

The case report article provides a good idea about POT especially because they are rare tumors and only a few cases have been reported.

 Article also writes all diagnosis methods and treatment outcomes and compared all cases of POT.

 Follow up of cases, I think, need a longer period than two years to decide no recurrence.

I have read this submission. I believe that I have an appropriate level of expertise to confirm that it is of an acceptable scientific standard.

## References

[1] AmerHHafedLIbrahimS: Case Report: A Primordial odontogenic tumor. *F1000Research*.2018;7: 10.12688/f1000research.14735.1 10.12688/f1000research.14735.1 PMC598914429904598

# Peer review: Referee response for version 1

**DOI:** 10.5256/f1000research.16032.r34183

**Published:** 2018-06-05

**Authors:** ‎Eman A. Abo Hager

**Affiliations:** 1 Oral and Dental patholology, Faculty of Dental Medicine, Al-Azhar University, Cairo, Egypt

This is an interesting case report describing in a full details a rare recently described mixed odontogenic tumor. I would prefer the authors to suggest further diagnostic test to differentiate peimordial odontogenic tumor from the aggressive odontogenic myxomas especially when cases unexpectedly appear radiographically multilocular radiolucent as in the present case.

I have read this submission. I believe that I have an appropriate level of expertise to confirm that it is of an acceptable scientific standard.

# Peer review: Referee response for version 1

**DOI:** 10.5256/f1000research.16032.r34185

**Published:** 2018-06-05

**Authors:** Marwa Mokbel ElShafei

**Affiliations:** 1 Oral Pathology, Misr International University (MIU) , Cairo, Egypt

The case report presented here is a well constructed case, the rarity and novelty of the lesion makes it useful for the clinicians and indeed, as stated, it may lead to a more conservative treatment and save the patient an aggressive treatment.

 The young age of the patient is interesting and gives a  probable reassurance for the parents as only a conservative treatment is needed.

 A clinical picture for the patient would have been useful if present and also a clinical picture after full recovery.

 A need to switch the magnification on the histopathologic pictures is to be done.

I have read this submission. I believe that I have an appropriate level of expertise to confirm that it is of an acceptable scientific standard.

# Peer review: Referee response for version 1

**DOI:** 10.5256/f1000research.16032.r34186

**Published:** 2018-05-23

**Authors:** Eman Abdelzaher

**Affiliations:** 1 Department of Pathology, Faculty of Medicine, Alexandria University, Alexandria, Egypt

This is a well-written case report describing a rare entity which was recently described.

 I would encourage the authors to add more histopathological details and elaborate on the discussion section mainly focusing on important differentiating points with histopathological mimics.

 Please click here for the annotated PDF of this article.

I have read this submission. I believe that I have an appropriate level of expertise to confirm that it is of an acceptable scientific standard.
